# Comparative Analysis of Mitochondrial Genomes in Distinct Nuclear Ploidy Loach *Misgurnus anguillicaudatus* and Its Implications for Polyploidy Evolution

**DOI:** 10.1371/journal.pone.0092033

**Published:** 2014-03-18

**Authors:** Xiaoyun Zhou, Yongyao Yu, Yanhe Li, Junjie Wu, Xiujie Zhang, Xianwu Guo, Weimin Wang

**Affiliations:** 1 College of Fisheries, Key Lab of Freshwater Animal Breeding, Ministry of Agriculture, Huazhong Agricultural University, Freshwater Aquaculture Collaborative Innovation Center of Hubei Province, Wuhan, P.R. China; 2 Laboratorio de Biomedicina Molecular, Centro de Biotecnología Genómica, Instituto Politécnico Nacional, Boulevard del Maestro esquina Elías Piña, Colonia Narciso Mendoza, Tamaulipas, Mexico; Institute of Hydrobiology, Chinese Academy of Sciences, China

## Abstract

*Misgurnus anguillicaudatus* has several natural ploidy types. To investigate whether nuclear polyploidy have an impact on mitochondrial DNA (mtDNA), the complete mitochondrial genomes (mitogenomes) of five distinct ploidy *M. anguillicaudatus* (natural diploid, triploid, tetraploid, pentaploid and hexaploid), which were collected in central China, were sequenced and analyzed. The five mitogenomes share the same gene arrangement and have similar gene size, base composition and codon usage pattern. The most variable regions of the mitogenome were the protein-coding genes, especially the *ND4L* (5.39% mutation rate). Most variations occurred in tetraploids. The phylogenetic tree showed that the tetraploid *M. anguillicaudatus* separated early from other ploidy loaches. Meanwhile, the mitogenomes from pentaploids, and hexaploids have the closest phylogenetic relations, but far from that of tetraploids, implying that pentaploids and hexaploids could not be formed from tetraploids, possibly from the diploids and triploids. The genus *Misgurnus* species were divided into two divergent inter-genus clades, and the five ploidy *M. anguillicaudatus* were monophyletic, which support the hypotheses about the mitochondrial introgression in loach species.

## Introduction

The cobitid loach *Misgurnus anguillicaudatus* (Cobitidae, Cypriniformes) is a small freshwater teleost that inhabits the muddy bottom of creeks, ponds, wetlands and paddy fields [1]. This species is widespread from China to Japan and other Southeast Asian countries, such as Indonesia and India [2]. The loach can be used as a traditional Chinese medicine or folk remedies for treatment of hepatitis, osteomyeitis, carbuncles, inflammations and cancers, as well as for patient's recovery from debilities caused by various pathogens and aging [3]. In the past two decades, commercial farming for *M. anguillicaudatus* occupies a significant position in freshwater fish aquaculture production in Asia [4].

The *M. anguillicaudatus* is full of interest to science because of the extensive ploidy variability in nature. In Japan, in addition to the most common bisexual diploid individuals (2n = 50), a relatively high abundant triploid loaches (3n = 75) can also be found in some localities [5]. Experimental crosses, including induced gynogenesis and inter-specific hybridization, shows that these triploid individuals were resulted from unreduced eggs fertilized by sympatric diploids [5]–[7]. In China, populations of both diploid and tetraploid (4n = 100) loaches have been frequently recorded [8]–[13]. A few triploid individuals have also been detected in several locations by measurement of the erythrocyte nucleus and determination of DNA content using flow cytometry [14]. The recent studies even detected rare pentaploid (5n = 125) [15] and hexaploid (6n = 150) [16] individuals in the Yangtze River basin. Owing to such natural variability at ploidy levels, *M. anguillicaudatus* can be a promising animal model for studying evolution of polyploid in fish genome.

The mitochondrial genome of vertebrates is a small and circular double-stranded molecule, usually about 15–18 kb in length, containing 13 protein-coding genes, 22 transfer RNA (tRNA) genes, two ribosomal RNA (rRNA) genes, and a putative control region [17], [18]. Due to its simple structure, maternal inheritance, fast evolutionary rate, and the resulting short coalescence time, mitochondrial DNA sequence data has been widely used in various studies from species identification (i.e., DNA barcoding) to molecular phylogeny [19]–[21]. As the mitochondrial DNA replication is controlled by enzymatic factors wholly encoded in the nuclear genome [22], and polyploidy can result in chromosomal rearrangements and gene loss [23], interlocus concerted evolution of ribosomal DNA repeats [24], unequal rates of sequences evolution of duplicated genes [25], and changes in DNA methylation [26], thus, nuclear polyploidization could has an impact on mitochondrial genome evolution.

Recent evidence demonstrates that, in addition to the creation of gene redundancy, polyploidization causes nuclear enlargement and increases the complexity of the processes that are involved in managing and partitioning chromosomes during cell division [27]. It has generally known that, ATP produced by the mitochondria is an essential requirement to drive the cell cycle. Inhibition of mitochondrial protein synthesis leads to G1 arrest and attenuates DNA replication [28], whereas increasing mitochondrial DNA copy number increases the transition from G1 to S and G2 to M, thereby accelerating the progression through the cell cycle [29], [30]. That's to say, mitochondrial processes play an active role in cell division and chromosomal replication [29]. Thus, changes in sets of nuclear chromosomes in polyploids may have effect on mtDNA sequences, which have been demonstrated in Yeast [31], but similar studies are lacking in vertebrates, including fish.

The polyploidy individuals (2n-6n) of *M. anguillicaudatus* exist in nature, which provides a good chance to study the relation between nuclear ployploidisation and its mitochondrial genome divergence. The mitogenomes of *M. anguillicaudatus* in China have been reported by He et al. [32] (DQ026434) and Zeng et al. [2] (AP010782). We compared the sequences of these two mitogenomes and found that the divergence is as large as 12%. Such a high genetic divergence might be related to the nuclear polyploidisation. In this study, the mitogenomes of the natural diploid, triploid, tetraploid, pentaploid and hexaploid *M. anguillicaudatus* were sequenced and compared. The phylogenetic relationship of five ploidy *M. anguillicaudatus* as well as 28 cobitid fishes using 13 protein-coding gene sequences was elucidated. The purpose of this study is to unravel whether nuclear polyploidy have an impact on the mitogenome of a species. On the other hand, the mtDNA analysis could infer some information on the evolution or formation of nuclear polyploidization in nature.

## Results and Discussion

### Mitogenome organization and composition

The mitogenomes of five-level ploidy *M. anguillicaudatus* were sequenced, annotated and submitted to the Genbank (see Table S2). Genome length, AT-richness and base composition of the five mitogenomes were compared in Table 1. These mitogenomes possessed a uniform gene arrangement, which are identical to other teleosts [37], [48], [49]. The lengths of the complete genome were 16644 bp for diploid, 16646 bp for triploid, 16645 bp for tetraploid, and 16643 bp for both pentaploid and hexaploid, respectively, corresponding to the typical length for fish mtDNA known to date [19], [20], [49], [50]. The overall base composition of these five mitogenomes is highly similar and exhibited the similar composition biases of A+T>G+C(Table 1), consistent with the lowest frequency for G among the four bases [51] and an A+T rich pattern of the vertebrate mitogenomes [50], [52].

**Table 1:** Mitochondrial genome sequences characteristic of five-level ploidy *M. anguillicaudatus.*

		Base composition in H strand				
Ploidy level	Size	A%	T%	C%	G%	(A+T)%
Diploid	16644 bp	29.79	28.35	25.56	16.30	58.14
Triploid	16646 bp	29.70	28.27	25.65	16.38	57.97
Tetraploid	16645 bp	29.60	28.26	25.81	16.53	57.86
Pentaploid	16643 bp	29.68	28.26	25.66	16.40	57.94
Hexaploid	16643 bp	29.66	28.24	25.68	16.42	57.90

**Figure 1:**
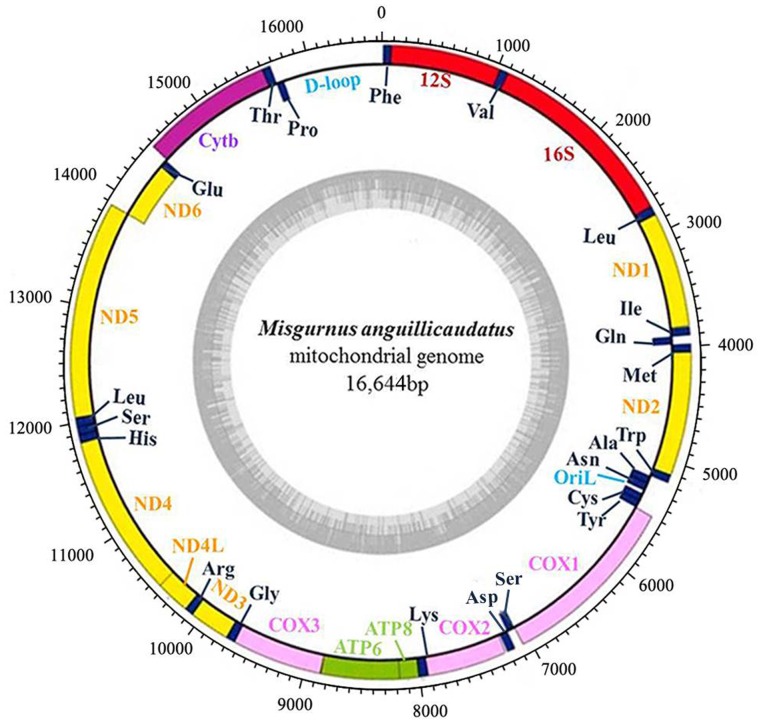
Gene map of *M. anguillicaudatus* mitochondrial genome. Genes encoded on the heavy or light strands are shown outside or inside the circular gene map, respectively. Inner ring indicated the GC content. All five ploidy *M. anguillicaudatus* individuals possessed a uniform gene arrangement and a similar gene size. The figure was initially generated with OGDRAW and modified manually.

Each mitogenome in the ploidy *M. anguillicaudatus* encodes the same 13 proteins, 22 tRNAs and 2 rRNAs with two noncoding regions — the control region (CR) and the origin of the light strand replication (*OriL*) — as found in other teleosts. These genes share the same direction and similar sequences in length. Except for one protein coding gene (*ND6*) and eight tRNAs (tRNA^Gln^, tRNA^Ala^, tRNA^Asn^, tRNA^Cys^, tRNA^Tyr^, tRNA^Ser^, tRNA^Glu^, and tRNA^Pro^) encoded on the L-strand, all other genes are encoded on the H-strand (Figure 1). Notably, gene overlapping, a common gene structure found in other vertebrate mitogenomes, was also detected between contiguous genes in five ploidy *M. anguillicaudatus* mitogenomes, and the length of these overlaps are generally being fixed [53]. For example, *ATPase 8* and *ATPase 6* genes overlapped by 10 bp, *ND4L* overlapping 7 bp with *ND4*, *ND5* overlapping 4 bp with *N*D6 (coded on the opposite strand)[18], [44], [49].

### Pairwise comparisons

**Figure 2:**
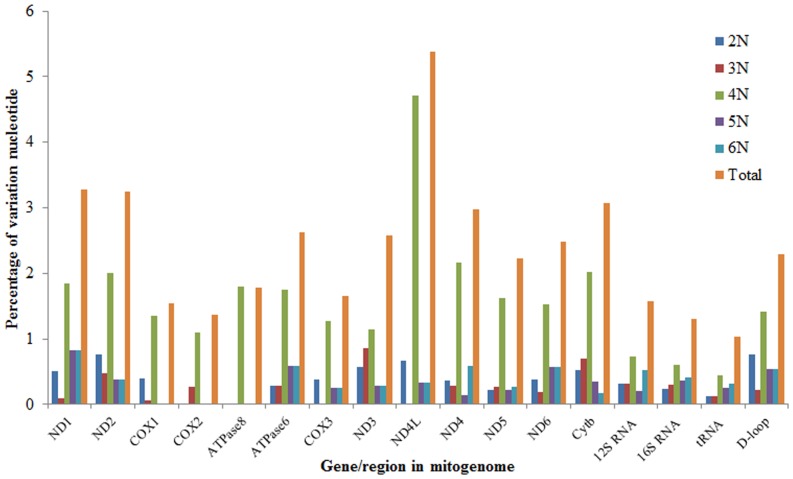
Percentage of nucleotide variation in different genes/regions of the five-level ploidy *M. anguillicaudatus*.

Pairwise comparisons of the mitogenome sequences were conducted. The similarities among the diploid, triploid, tetraploid, pentaploid, and hexaploid *M. anguillicaudatus* varied from 98.3% to 99.8%. The lowest similarities (98.3%) occurred between the tetraploids and pentaploids as well as the tetraploids and hexaploid. The highest similarities (99.8%) occurred between the pentaploids and hexaploids. The divergence of the five mitogenomes ranged from 0.15% between the pentaploids and hexaploids to 1.71% between the tetraploids and pentaploids. A total of 453 single nucleotide polymorphisms (SNPs) were found, accounting for 2.72% of the total sites. The most variable regions of the genome are the protein-coding regions, especially the *ND4L* (5.39% genetic divergence), followed by noncoding intergenic spacer regions (2.21%). The tRNA genes are characterized by the lowest percentage variability (1.03%) (Figure 2). In addition, of the 453 variable site detected, 239 occurred in tetraploids, while only 61, 44, 49, 60 were present in diploids, triploids, pentaploids and hexaploids, respectively. Furthermore, of all genes or regions, the tetraploids displayed the highest genetic divergence (Figure 2).

### Comparative analyses of protein-coding genes

**Figure 3:**
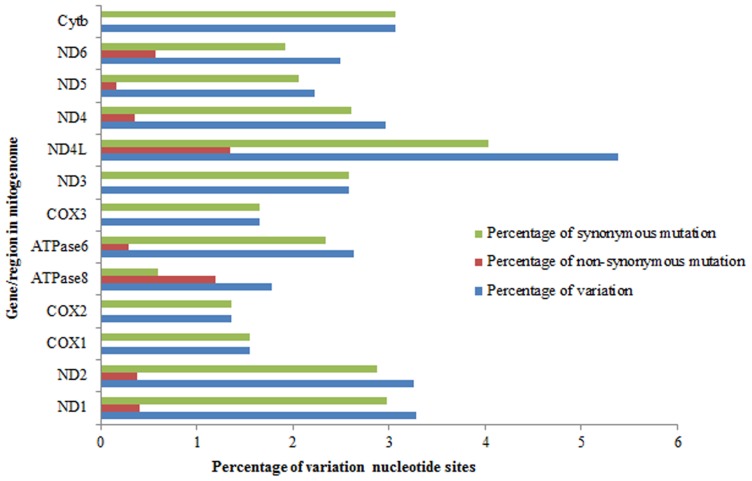
Overall, synonymous and non-synonymous mutations for different genes/regions of the five-level ploidy *M. anguillicaudatus*.

Total 11,472 bp of the 13 mitochondrial protein-coding genes, 290 variable sites (accounting for 2.53%) were observed among five mitogenomes. As reported in other vertebrate mtDNAs, most nucleotide substitutions were located at the third codon position, resulting in synonymous mutations [54], [55] (Figure 3). Therefore, of the 3842 amino acids deduced from the nucleotide sequences of the 13 protein-coding genes, only 27 amino acid (accounting for 0.70%) replacements occurred within the five ploidy levels. Among the 13 protein-coding genes, the *ND4L* gene displayed the highest variability (5.39%), with four synonymous mutations (1.35%) and 12 non-synonymous mutations (4.04%). While the lowest variability was found in *COX2* gene, with only 10 variable sites (1.36%) detected, all of which were synonymous mutations. No non-synonymous mutations were also observed in *COX1*, *COX3*, *ND3*, and *Cytb* genes. On the other hand, within the 290 variable sites, 173 (accounting for 59.66%) occurred in the tetraploids, indicating a relative genetic distance between tetraploids and other ploidy varieties. Besides, the high level of genetic divergence in protein-coding genes may indicate that different ploidies were under divergent selection, gene flow and/or mitochondrial introgression brought different haplotypes together in single individuals. However, it needs more samples from wider geographical regions and more evolutionarily divergent lineages from different evolutionary depth to clarify.

The size and encoding direction of 13 protein-coding genes in five mitogenome sequences were almost completely the same. All 13 protein-coding genes initiate with the traditional ATG start codon except for *COX1*, which begins with GTG as in all bony fishes [18]. However, the termination codons of 13 protein-coding genes include seven TAA, one TAG (*ND5*), and five incomplete stop codons TNN (*ND2*, *COX3*, *ND3*, and *Cytb*) or TAN(*ND4*). Such immature stop codons are common among vertebrate mitogenomes and it appears that TAA stop codons are created via post-transcriptional polyadenylation [50], [56], [57]. The most abundant amino acid residue encoded by the 13 protein-coding genes was leucine (accounting 11.72% to 11.85%), whereas the rarest was cysteine (accounting 0.76% to 0.79%).

### Variation in ribosomal and transfer RNA genes

All mitogenomes of ploidy *M. anguillicaudatus* contain two rRNA sub-units, 12S and 16S, which are separated by tRNA^Val^ as in the other vertebrates [55]. All five mitogenomes have the same length of 12S RNA with 953 bp. However, the length of 16S RNA varied: triploids and tetraploids have 1679 bp, but one deletion was observed in diploid and three same nucleotide deletions were occurred in pentaploid and hexaploid, leading to 1678 bp in diploid, whereas 1676 bp in pentaploids and hexaploids. Length variation in 16S RNA has been also reported in other species such as *Cyprinus carpio* and *C. auratus* [58], [59]. Among five ploidy mitogenomes, 37 variable sites were detected (15 in 12S RNA and 22 in 16S RNA). However, 20 of those occurred in tetraploids.

The tRNAs genes are the lowest variability region in the mitogenomes. Out of 1558 nucleotide sites (1557 in diploids) of the 22 tRNA genes, only 16 variable sites (accounting for 1.03%) were detected, and five of them were observed in tetraploids. No variability was observed in the anticodon positions. In addition, there are overlaps between adjacent tRNA genes as seen in the mitogeneomes of other bony fishes [60], [61]. For example, two nucleotides overlap between tRNA^Ile^ and tRNA^Gln^, and two between tRNA^Thr^ and tRNA^Pro^. Of the 22 tRNA genes, 21 could be folded into the classic cloverleaf secondary structure by tRNAscan-SE1.2.1 [40], while the tRNA for serine with the anticodon AGY (tRNA^Ser(AGY)^) lacks a DHU arm, which is a common finding in all vertebrates [62].

### Comparison of non-coding regions

The major noncoding region (D-loop), located between tRNA^Pro^ and tRNA^Phe^ genes, is 918 bp in length (917 bp in tetraploids). As in other fish species, several conserved domains and motifs that are associated with the initiation of DNA replication and transcription were recognized by multiple homologous sequence alignment and recognition site comparison. The first domain is the terminal associated sequences (TAS) involved in the termination of H-strand synthesis, located at the 5′ end of D-loop region. Four copies of the conversed motif TACAT and its complement ATGTA in this domain were detected in each ploidy level *M. anguillicaudatus* and form a thermostable “hairpin” structure to regulate mitochondrial gene replication [61], [63]. The second domain is the central conserved-blocks, containing CSB-D, CSB-E and CSB-F. The third domain consists of three conserved sequence blocks 1-3, found in the 3′ end of D-loop region, appeared to be involved in positioning RNA polymerase, both for transcription and for priming replication[64]–[66]. In comparison with results reported earlier by Lee et al. [67] and Yan et al. [66] as well as other cobitid fishes, the consensus sequence of the conserved motifs in *M. anguillicaudatus* were identified and exhibited in Table 2.

**Table 2:** The conserved consensus sequence in D-loop region of the five-level ploidy *M. anguillicaudatus* based on the structure of the D-loop region in other fishes.

Conserved motifs	Consensus sequences
TAS	TACAT-ATGTATTATCACC
CSB-F	ATGTAGTAAGAAACCACCAACCA
CSB-E	ATGATAGG-TCAGGGACAA
CSB-D	GTGAACTATTACTGGCATCTGG
CSB-1	TGTGATTGAATG-T—AAAGACATAA
CSB-2	CAAACCCCCCTACCCCC
CSB-3	TGTCAAACCCCGAAACCAA

**Figure 4:**
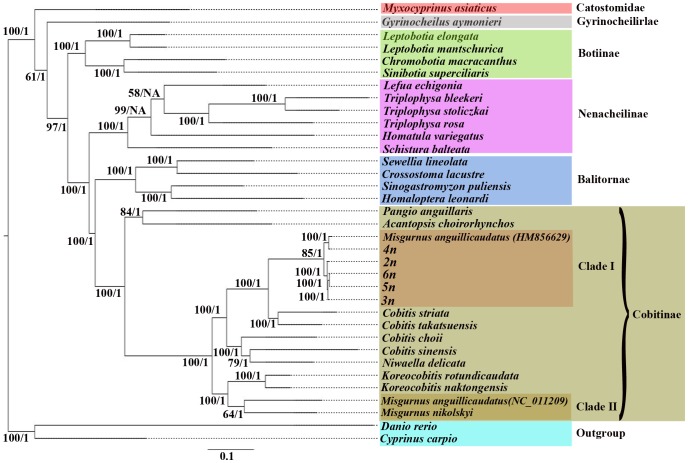
Phylogenetic analyses of five-level ploidy *M. anguillicaudatus* and other 28 Cobitoidea species. The species and their NCBI Accession No. were listed in Table S2. The phylogenetic analyses were conducted based on the concatenated 13 mitochondrial protein-coding genes with maximum likelihood (ML) and Bayesian inference (BI) methods. Numbers on the nodes represent support values inferred from ML (left) bootstrap and BI (right) probability analyses, respectively (only values above 50 of bootstrap value or 0.50 of Bayesian posterior probability are shown). NA indicates the nod by BI is not essentially identical to that of the ML tree.

It is widely accepted that the noncoding region evolves faster and characterized by the higher percentage variability than protein-encoding genes [49], [53], [68]. However, in this study, the variation analysis among five distinct ploidy *M. anguillicaudatus* results showed that, only 2.29% of the nucleotide sites were variable, which was lower than most of the protein-encoding genes, such as the *ND1* (3.28%), *ND2* (3.25%), *ATPase6* (2.63%), *ND3* (2.58%), *ND4L* (5.39%), *ND4* (2.97%), and *Cyt b* genes (3.07%). The results indicated a slow rate of evolution in the D-loop region in *M. anguillicaudatus*. Slower evolution of the D-loop region than that of protein-encoding gene was also found in some avian species [69] and very common in Cypriniformes[68]. Researchers considered that, although as a noncoding region, the D-loop region contains sequences related to termination of H-strand replication, the origin of H-strand, and promoters of transcription to both L- and H-strand [70]–[74]. Besides, many conserved sequence blocks identified suggest that unknown functions could exist. These known and unknown functions put the D-loop region under high evolutionary pressure and lead to the slow rate of substitution [68]. The sequences of D-loop region have been extensively used for phylogenic analysis. The low variations in this region thus could affect the phylogeny analysis for this group of fish. We thus did the phylogeny analysis with both ML and BL methods based on the D-loop region and the trees exhibited similar phylogenetic topologies with the results constructed using the 13 protein-coding genes in Cobitinae (Figure S2, Figure 4). It indicates that although D-loop region contains low variability in contrast to the protein-coding genes, the variations in this region is still informative for phylogenic analysis for this group.

In all five distinct ploidy *M. anguillicaudatus*, a small non-coding region of 30 bp, the putative origin of light strand replication (oriL), is located in the cluster of the tRNA^Trp^-tRNA^Ala^-tRNA^Asn^-tRNA^Cys^-tRNA^Tyr^ region (the WANVY region) between the tRNA^Asn^ and tRNA^Cys^ genes [75]. This region can be folded into a stable stem-loop secondary structure with 12 bp in the stem and 15 bp in the loop. Thus, it may be the reason why no variation can be detected in this region among our sequenced mitogenomes.

### Phylogenetic relationships

To understand the phylogenetic relationships of the diploid and polyploid species and to estimate the origin of polyploidy in the evolution of Cobitidae, the concatenated nucleotide sequences of the 13 protein-coding genes of each distinct ploidy *M. anguillicaudatus* obtained in this study, as well as previously reported 28 cobitidae species were used for phylogenetic analyses. The trees constructed by ML and BI methods exhibited similar phylogenetic topologies (Figure 4).

As expected, the species from Cobitinae, Balitornate, Nemacheilinae, and Botiinae were monophyletic and form one clade, which were in agreement with the phylogenetic results based on the morphological data [76] and molecular biology [68]. However, within the Cobitinae, the genus *Misgurnus* was not in monophyletic group but divided into two divergent clades, I and II. The Clade I consisted of five-level ploidy *M. anguillicaudatus* obtained in this study and the *M. anguillicaudatus* (HM856629) reported by Zeng et al.[2], and then clustered with the genus *Cobitis* with high bootstrap supports (Bootstrap value  =  100, BP  =  1). While in the Clade II, the *M. anguillicaudatus* (DQ026434) reported by He et al. [32] was clustered with *M. nikolskyi*, and then clustered with genus *Koreocobitis*. The average sequence divergence between these two clades was 13.83%.

Two genetically divergent mitochondrial clades in *M. anguillicaudatus* appeared as widespread phenomenon and have previously been identified by phylogeographic studies using mtDNA sequences and nuclear genes. Based on the control region [77] and the cytochrome *b* gene sequences [78], the Japanese populations of *M. anguillicaudatus* were divided into two distinct major clades, of which one was closely related to the European *Misgurnus fossilis* and the East Asian *Paramisgurnus dabryanus*, while the other was distantly *Misgurnus* monophyletic [1]. Another molecular phylogenetic study of Cobitidea species using nuclear DNA gene sequences revealed that *Misgurnus* and its relatives (*Paramisgurnus* and *Koreocobitis*) as well as *Cobitis* (excluding *Cobitis misgurnuoides*) and its relatives (Niwaella, Iksookimia and *Kichulchoia*) were reciprocally monophyletic [79]. However, with mtDNA data, the majority of the samples of East Asian *Misgurnus* representing at least five species from Russia, China, Korea and Japan, were included in the *Cobitis* clade. This discrepancy between mitochondrial and nuclear gene trees was explained to be a result of hybridization and subsequent mtDNA introgression occurred between an ancestral species of *Cobitis* and an ancestral species of *Misgurnus* [1], [79]. By further phylogeographic analyses of these two distinct clades *M. anguillicaudatus* based on mtDNA cytochrome *b* sequences, Kitagawa et al. [1] supported this mtDNA introgression hypothesis and proposed the zoogeographic historical process of *M. anguillicaudatus*, that is, after the hybridization and mtDNA introgression, the *M. anguillicaudatus* with introgressed mtDNA stretched over most of East Asia, including China, Japan and Korea. For the two clades, *M. anguillicaudatus* identified in previously studies, one corresponding to the individuals carrying introgressed mtDNA from the genus *Cobitis*, while another one corresponds to the relic of *M. anguillicaudatus* with non-introgressed mtDNA. Therefore, we supposed that, the five distinctly ploidy individuals studied in the present study and the sample from Poyang Lake [2] could be considered as the introgressed mtDNA type because they were closely related to the *Cobitis* species, whereas the *M. anguillicaudatus* reported by He et al.[32] should be the non-introgressed mtDNA type.

From the information of mitogenome, it could get some implication on the evolution of nuclear polyploidisation. This phylogenetic tree showed that the tetraploid *M. anguillicaudatus* separated early from other ploidy loaches. The recent molecular phylogenetic study of Cobitidae species using nuclear gene [79], [80] as well as the mitochondrial gene sequences [1], [77]–[80] suggested an ancient hybridization events occurred early in the evolutionary history of *Misgurnus*. The interspecific hybridization tends to trigger the development of asexuality and subsequently, polyploidisation, as recognized well in the spined loaches [81], [82], which is exactly consistent with our results. In fact, the tetraploid mitogenome accumulated mutations more than half the total mutations of five genomes sequenced in the present study. It is thus reasonable that the natural tetraploidy *M. anguillicaudatus* in Yangtze River could be also the result of this historic hybridization event in the early evolution of *M. anguillicaudatus*. Meanwhile, the mitogenomes from pentaploids, and hexaploids have the closest phylogenetic relations, but far from that of tetraploids, implying that pentaploids, and hexaploids could not be formed from tetraploids, possibly from the diploids and triploids.

## Conclusions

We reported the comparisons of mitogenome sequences of the five-level ploidy *M. anguillicaudatus*: diploids, triploids, tetraploids, pentaploids, and hexaploids. The results demonstrated that the polyploidy have not clear impact on the content, organization or sequence of natural Cobitoidea species' mtDNA. However, the mitogenome analysis under five-level nuclear ploidy background still produced some inference on the evolution of nuclear polyploidisation. In addition, the genus *Misgurnus* includes two major mtDNA clades support the recently developed hypotheses on the mitochondrial introgression in loach species.

## Materials and Methods

### Ethics statement

Before each handling, the fish were anaesthetizing with tricaine methanesulfonate (MS-222) at 100 mg/L. All the experimental procedures involving fish were approved by the institution animal care and use committee of Huazhong Agricultural University.

### Samples and DNA extraction

The *M. anguillicaudatus* individuals were collected from the Yangtze River basin, China. Diploid, triploid, tetraploid, and pentaploid individuals were obtained from Liangzi Lake area [15], while the hexaploids were collected from the Diaocha Lake area[16](Figure S1). The sites are located in an open, abandoned field and no specific permit is required for the described field studies. Loach species was identified according to Chen & Zhu [33], while the ploidy level of *M. anguillicaudatus* was determined by using flow cytometer (BD FACS Calibur, USA)[34]. Subsequently, a small portion of the caudal fin from each sample was taken and immediately preserved in 95% ethanol. Total genomic DNA was extracted from fin tissues using the ammonium acetate method [35]. The concentration and purity of the extracted DNA were measured using the NanoDrop 2000 (Thermo Scientific, Wilmington, DE, USA).

### PCR amplification and sequencing

The mitogenomes of *M. anguillicaudatus* were amplified using long PCR method [36]. Two sets of fish-versatile primer pairs (S-LA-16S-L+H15149-CYB and L12321-Leu+S-LA-16S-H) (Table S1) were used to amplify almost the entire mitogenome for two reactions [2], [37], [38]. The reactions were performed in an Eppendorf Thermal Cycler (Berlin, Germany) with 25 μl reaction volume containing 10× LA PCR buffer II (Mg^2+^), 1.25 mM of dNTPs, 0.5 mM of each primer, 1.25 U of LA *Taq* polymerase (Takara), approximately 100 ng of template DNA. The thermal cycle profile was: pre-denaturation at 94°C for 1 min, followed by 30 cycles of 98°C for 10 s, 68°C for 16 min, and finally with 72°C for 10 min. The long PCR products were sequenced by primer walking strategy, with the primer pairs NQ1-NQ18, which were designed according to the reported complete mitogenome sequences of *M. anguillicaudatus* [2], [32] (Table S1). The sequencing was conducted by Sangon Biotech (Shanghai, China) Co., Ltd.

### Gene annotation and sequence analysis

The sequence fragments obtained were edited in the Seqmen program (DNAstar, Madison, WI, USA) for contig assembly to obtain the complete mitogenome sequences. Annotation of protein-coding and ribosomal RNA genes, and definition of their respective gene boundaries were carried out with DOGMA software [39]. The tRNAs and their secondary structures were identified by tRNAscan-SE 1.21 software[40]. Putative origin of light strand replication (*OriL*), control region (CR), and conserved motifs were identified via sequence homology.

Sequences were aligned using the Clustal W [41]. Pairwise distances of the nucleotide sequences of the mitogenomes were estimated using Kimura's two-parameter method. The protein-coding gene alignment at the nucleotide level was based on the information provided by the protein alignment. Base composition and pattern (models of substitution, transition-to-transvertion [ts/tv] ratios, base composition distances) of evolution for each gene or each protein-coding gene, as appropriate, were determined with MEGA 5.0 [42]. The numbers of variable sites, nucleotide diversity, synonymous and non-synonymous distance among the five-level ploidy *M. anguillicaudatus* mitogenomes were estimated with DnaSP v5.0 [43]. The nucleotide variations in each genes/regions of the five-level ploidy *M. anguillicaudatus* were calculated by percent of variable sites.

### Phylogenetic analyses

As protein-coding genes are informative in inferring species phylogeny [44], the nucleotide sequences of mitochondrial proteins from five ploidy *M. anguillicaudatus* mitogenomes were sequenced in this study. The previously reported mitogenome sequences of 28 cobitidae species and two out-group species (*Danio rerio* and *Cyprinus carpio*) were downloaded from GenBank (Table S2). The nucleotide sequences of 13 mitochondrial protein-coding genes were separately aligned using Clustal X [45], the gaps and ambiguous areas were excluded manually. The phylogenetic analysis were performed based on 13 concatenated mitochondrial protein-coding genes by the maximum likelihood (ML) and Bayesian inference (BI) methods, using MEGA version 5.0 [42] and MrBayes 3.1.2 [46], respectively.

The jModeltest program [47] was used to determine the best fitting models of nucleotide substitution. The Akanke's Information Criterion (AIC) indicated that the GTR+I+G model are the most appropriate for the dataset. In the BI analyses, the following settings were applied: number of Markov chain Monte Carlo (MCMC) generations  =  three million, sampling frequency  =  1000, burn-in  =  250. The robustness of the resultant ML tree was evaluated using bootstrap probabilities calculated from nonparametric bootstrap analyses with 500 pseudo-replications.

## Supporting Information

Figure S1 Map of China showing the sampling location at Liangzi Lake area (30°12′55″N 114°30′7″E) and Diaochahu area (113°43′18.5″ E, 30°39′44.6″ N), Hubei province, China.
(PDF)

Figure S2 Phylogenetic analyses of five-level ploidy *M. anguillicaudatus* and other 28 Cobitoidea species using the D-loop region.
(PDF)

Table S1 Primers designed for amplifying mitochondrial genome of *Misgurnus anguillicaudatus.*
(PDF)

Table S2 List of species used in the phylogenetic analyses and their references.
(PDF)
