# Supplementary material for: Comparative Analysis of Mitochondrial Genomes in Distinct Nuclear Ploidy Loach Misgurnus anguillicaudatus and Its Implications for Polyploidy Evolution
Source: PLoS One. 2014 Mar 18;9(3):e92033. doi: 10.1371/journal.pone.0092033 (PMC3958399; doi:10.1371/journal.pone.0092033)
Supplement: Figure S1 — Map of China showing the sampling location at Liangzi Lake area (30°12′55″N 114°30′7″E) and Diaochahu area (113°43′18.5″ E, 30°39′44.6″ N), Hubei province, China. (PDF) [file pone.0092033.s001.pdf]

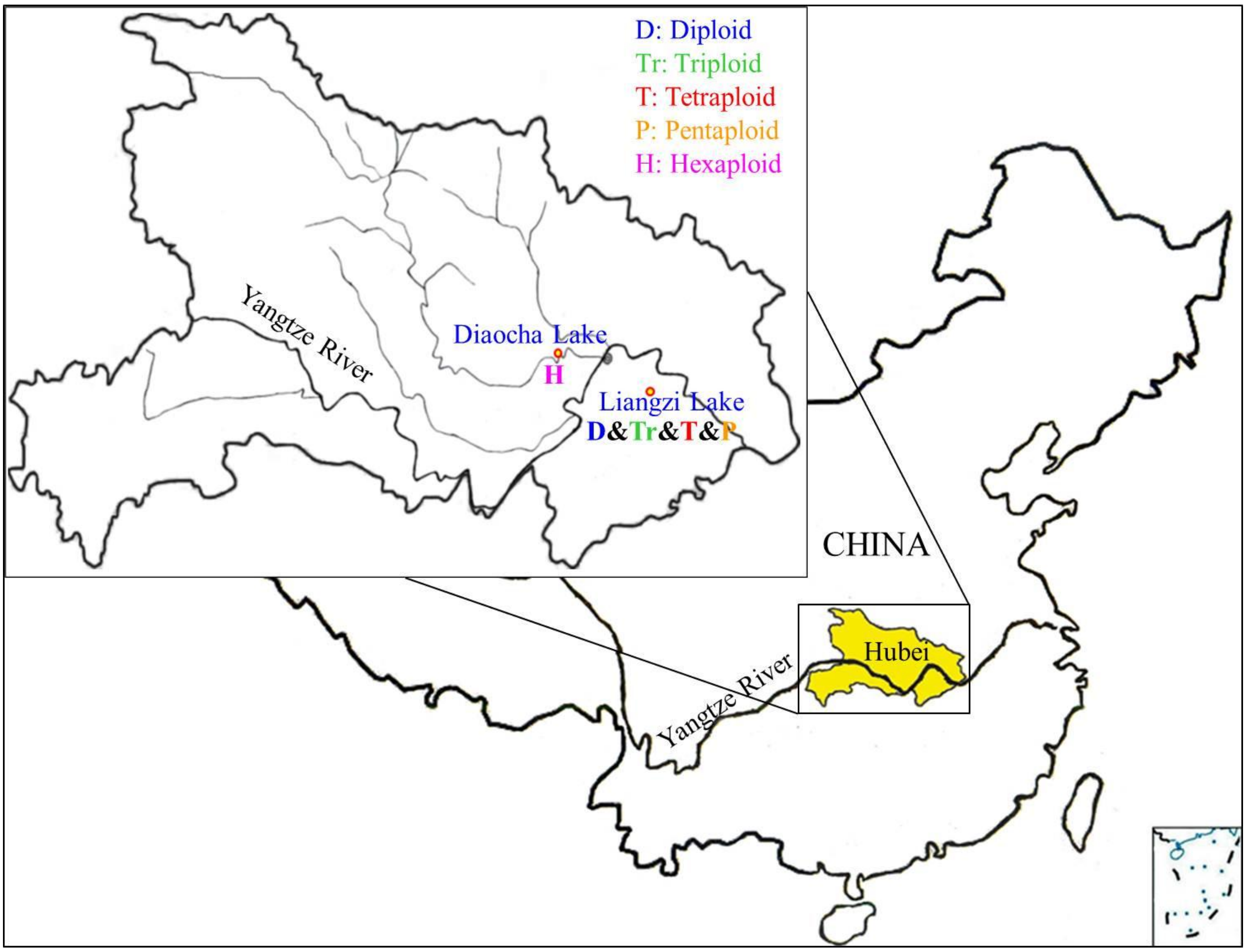

Figure S1 Map of China showing the sampling location at Liangzi Lake area (30 °12'55"N 114 °30'7"E) and Diaochahu area (113°43′18.5″ E, 30°39′44.6″ N), Hubei province, China.
