# Supplementary figures and images for: Comparative Analysis of Mitochondrial Genomes in Distinct Nuclear Ploidy Loach Misgurnus anguillicaudatus and Its Implications for Polyploidy Evolution
Source: PLoS One. 2014 Mar 18;9(3):e92033. doi: 10.1371/journal.pone.0092033 (PMC3958399; doi:10.1371/journal.pone.0092033)

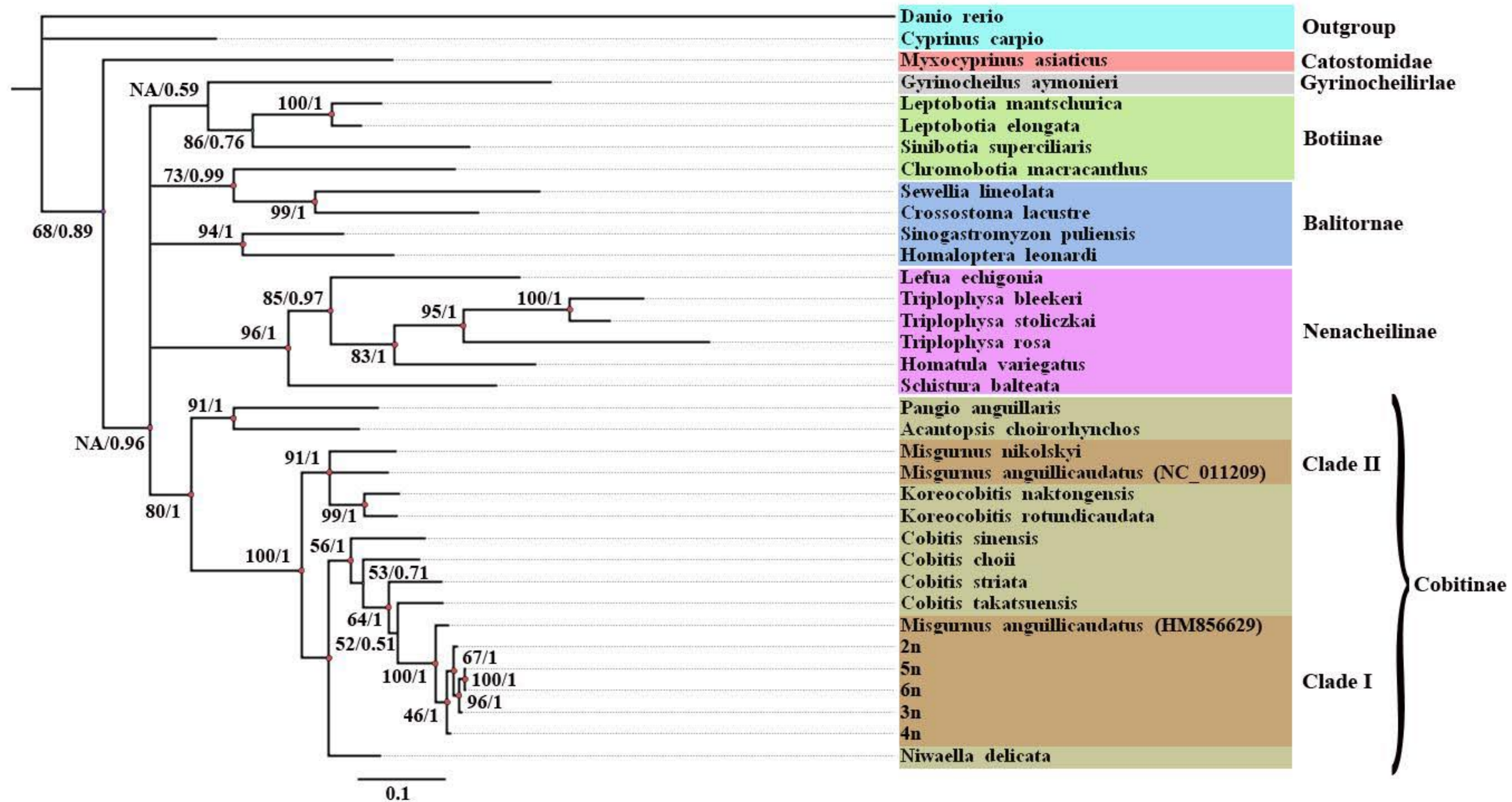

Supplement: Figure S2 — Phylogenetic analyses of five-level ploidy M. anguillicaudatus and other 28 Cobitoidea species using the D-loop region. (PDF) [file pone.0092033.s002.pdf]
