# Supplementary material for: Comparative Analysis of Mitochondrial Genomes in Distinct Nuclear Ploidy Loach Misgurnus anguillicaudatus and Its Implications for Polyploidy Evolution
Source: PLoS One. 2014 Mar 18;9(3):e92033. doi: 10.1371/journal.pone.0092033 (PMC3958399; doi:10.1371/journal.pone.0092033)
Supplement: Table S1 — Primers designed for amplifying mitochondrial genome of Misgurnus anguillicaudatus. (PDF) [file pone.0092033.s003.pdf]

Table S1 Primers designed for amplifying mitochondrial genome of *Misgurnus anguillicaudatus*

Forward	Sequence (5'-3')	Reverse	Sequence (5'-3')
Long PCR primers			
S-LA-16S-H	TGCACCATTRGGATGTCCTGATC CAACATC	L12321-Leu	GGTCTTAGGAACCAAAAACTCTT GGTGCAA
S-LA-16S-L	CGATTAAAGTCCTACGTGATCTG AGTTCAG	H15149-CYB	GGTGGCKCCTCAGAAGGACATTT GKCCTCA
Internal primers			
NQ1F	CACTGAAGATGCTAAGATGG	NQ1R	TCTCTGCCTGTTGTATGC
NQ2F	TTACACCGAGAAGACATCC	NQ2R	AGTTCCATTATCCGCTAAGT
NQ3F	CAGTGACCACAAGTTCAAC	NQ3R	GCATATTCAGCCAGGAAGA
NQ4F	TCTCTAGCCTTGCCGTATA	NQ4R	GTTGATAGGATTAGACCTGTTG
NQ5F	ATAGCACAGCAGCATCAC	NQ5R	GCAGTTCCAACCATTCCA
NQ6F	AACTTAGACCAAGAGCCTTC	NQ6R	CACGAGTATCAACATCTATTCC
NQ7F	ACTGCCGTTCTTCTTCTAC	NQ7R	AGCCTAAGTCCTCATAGTCA
NQ8F	ATAACCACTCTGCCACTTC	NQ8R	CGATTGATTAGTCAGCCTTG
NQ9F	CGTTCCACTTGAGCACTT	NQ9R	ATGGTCAGAAGAAGCAGAAT
NQ10F	GTCTATTCATTCGTCCATTAGC	NQ10R	GCATTGTAGGAGATTGAGGTT
NQ11F	AAGACCGTGGTTCAACTC	NQ11R	TGTGTTCGCTCGTAAGTG
NQ12F	TGTTCACCTCTGACTACCTA	NQ12R	CATCTGCTCGTCCGTATC
NQ13F	CAACAACTCTAATCCTCTCCT	NQ13R	CCAGCAATAATACTTCCTCAG
NQ14F	CTGGCATTCCTTCACATCT	NQ14R	TTCTTCAAGTCACTGGTCTC
NQ15F	CTGCCACCACTAATCCTAA	NQ15R	GCTCACTCTAATGCCTTGT
NQ16F	GCCACTATTCTACATCTACTCT	NQ16R	GCATAAGTCAACACCTACTG
NQ17F	AACAAGGCATTAGAGTGAG	NQ17R	GTATGACAGCCAAGAGGT
NQ18F	GATACCAGTAGAACATCCAT	NQ18R	GCCCTCTTATCCCTAACTA
