# Supplementary material for: Comparative Analysis of Mitochondrial Genomes in Distinct Nuclear Ploidy Loach Misgurnus anguillicaudatus and Its Implications for Polyploidy Evolution
Source: PLoS One. 2014 Mar 18;9(3):e92033. doi: 10.1371/journal.pone.0092033 (PMC3958399; doi:10.1371/journal.pone.0092033)
Supplement: Table S2 — List of species used in the phylogenetic analyses and their references. (PDF) [file pone.0092033.s004.pdf]

Table S2 List of species used in the phylogenetic analyses and their references.

Species	Accession No.	References
Danio rerio	NC_002333	Broughton et al. 2001
Cyprinus carpio	NC_001606	Chang et al. 1994
Myxocyprinus asiaticus	AY986503	Peng et al.2006
Gyrinocheilus aymonieri	DQ026432	He et al.2008
Lefua echigonia	AB054126	Saitoh et al.2003
Homaloptera leonardi	AB242165	Saitoh et al.2006
Misgurnus nikolskyi	AB242171	Saitoh et al.2006
Pangio anguillaris	AB242168	Saitoh et al.2006
Schistura balteata	AB242172	Saitoh et al.2006
Leptobotia mantschurica	AB242170	Saitoh et al.2006
Chromobotia macracanthus	AB242163	Saitoh et al.2006
Acantopsis choirorhynchos	AB242161	Saitoh et al.2006
Cobitis striata	AP010782	Saitoh et al.2010
Cobitis takatsuensis	AP009306	Saitoh et al.2010
Niwaella delicata	AP009308	Saitoh et al.2010
Crossostoma lacustre	AP010774	Saitoh et al.2011
Homatula variegatus	JX144893	
Cobitis choii	EU656112	Kim et al.2008
Koreocobitis naktongensis	JN607252	Kim & Bang 2012
Koreocobitis rotundicaudata	JN607253	Kim & Bang 2012
Leptobotia elongata	JQ230103	Li et al.2012
Cobitis sinensis	AY526868	Wang et al. 2011
Sinibotia superciliaris	JX155735	Ye et al.2013
Sinogastromyzon puliensis	FJ605359	
Sewellia lineolata	AP011292	Tang et al.2010

Triplophysa bleekeri	JX135578	Tang et al.2013
Triplophysa rosa	JF268621	Wang et al.2012
Triplophysa stoliczkai	JQ663847	Li et al.2013
Misgurnus anguillicaudatus (*WH*)	NC_011209	He et al.2008
Misgurnus anguillicaudatus (*PY*)	HM856629	Zeng et al.2012
Misgurnus anguillicaudatus (*Diploid*)	KC884745	This study
Misgurnus anguillicaudatus (*Triploid*)	KC823274	This study
Misgurnus anguillicaudatus (Tetraploid)	KC762740	This study
Misgurnus anguillicaudatus (Pentaploid)	KC881110	This study
Misgurnus anguillicaudatus (Hexaploid)	KC734881	This study

Reference

1. Broughton RE, Milam JE, Roe BA (2001) The complete sequence of the zebrafish (*Danio rerio*) mitochondrial genome and evolutionary patterns in vertebrate mitochondrial DNA. Genome Research 11:1958-1967.
2. Chang Y, Huang F, Lo T (1994) The complete nucleotide sequence and gene organization of carp (*Cyprinus carpio*) mitochondrial genome. Journal of Molecular Evolution 38: 138-155.
3. Peng Z, Wang J, He X (2006) The complete mitochondrial genome of the helmet catfish *Cranoglanis bouderius* (Siluriformes: Cranoglanididae) and the phylogeny of otophysan fishes. Gene 376: 290-297.
4. He S, Gu X, Mayden RL, Chen WJ, Conway KW, et al. (2008) Phylogenetic position of the enigmatic genus *Psilorhynchus* (Ostariophysi: Cypriniformes): evidence from the mitochondrial genome. Molecular Phylogenetics and Evolution 47:419-425.
5. Saitoh K, Miya M, Inoue JG, Ishiguro NB, Nishida M (2003) Mitochondrial genomics of ostariophysan fishes: perspectives on phylogeny and biogeography. Journal of Molecular Evolution 56:464-472.
6. Saitoh K, Sado T, Mayden RL, Hanzawa N, Nakamura K, et al. (2006) Mitogenomic evolution and relationships of the Cypriniformes (Actinopterygii: Ostariophysi): the first evidence toward resolution of higher-level relationships of the world's largest freshwater fish clade based on 59 whole mitogenome sequences. Journal of Molecular Evolution 63:826-841.
7. Saitoh K, Chen WJ, Mayden RL (2010) Extensive hybridization and tetrapolyploidy in spined loach fish. Molecular Phylogenetics and Evolution 56:1001-1010.

8. Saitoh K, Sado T, Doosey MH, Bart HL, Jr Inoue JG, et al. (2011) Evidence from mitochondrial genomics supports the lower Mesozoic of South Asia as the time and place of basal divergence of cypriniform fishes (Actinopterygii: Ostariophysi). Zoological Journal of the Linnean Society 161:633-662.
9. Kim KY, Lee SY, Bang IC, Nam YK (2008) Complete mitogenome sequence of an endangered freshwater fish, *Iksookimia choii* (Teleostei; Cypriniformes; Cobitidae). Mitochondrial DNA 19:438-445.
10. Kim KY, Bang IC (2012) Phylogeny and speciation time estimation of two *Koreocobitis* species (Teleostei; Cypriniformes; Cobitidae) endemic to Korea inferred from their complete mitogenomic sequences. Genes Genomics 34:35-42.
11. Li P, Yang C, Tu F, Liu G (2012) The complete mitochondrial genome of the Elongate loach *Leptobotia elongata* (Cypriniformes: Cobitidae). Mitochondrial DNA 23:352-354.
12. Wang JL, Shen T, Ju JF, Yang GA (2011) The complete mitochondrial genome of the Chinese longsnout catfish *Leiocassis longirostris* (Siluriformes: Bagridae) and a time-calibrated phylogeny of ostariophysan fishes. Molecular Biology Reports 38: 2507-2516.
13. Ye H, Huang Y, Wang J, Peng Z, Zhang Y (2013) Determination and comparison of two complete mitochondrial genomes of golden Chinese loach, *Sinibotia superciliaris* (Teleostei, Cypriniformes). Mitochondrial DNA 24:34-36.
14. Tang KL, Agnew MK, Hirt MV, Sado T, Schneider LM, et al. (2010) Systematics of the subfamily Danioninae (Teleostei: Cypriniformes: Cyprinidae). Molecular Phylogenetics and Evolution 57:189-214.
15. Tang Q, Huang Y, Wang J, Huang J, Wang Z, et al. (2013) The complete mitochondrial genome sequence of *Triplophysa bleekeri* (Teleostei, Balitoridae, Nemacheilinae). Mitochondrial DNA 24:25-27.
16. Wang J, Tang Q, Wang Z, Zhang Y, Wu Q, et al. (2012) The complete mitogenome sequence of a cave loach *Triplophysa rosa* (Teleostei, Balitoridae, Nemacheilinae). Mitochondrial DNA 23:366-368.
17. Li T, Gao C, Cui Y, Xie Q, Bu W (2013) The Complete Mitochondrial Genome of the Stalk-Eyed Bug *Chauliops fallax* Scott, and the Monophyly of Malcidae (Hemiptera: Heteroptera). PLoS ONE 8(2): e55381.
18. Zeng L, Wang J, Sheng J, Gu Q, Hong Y (2012) Molecular characteristics of mitochondrial DNA and phylogenetic analysis of the loach (*Misgurnus anguillicaudatus*) from the Poyang Lake. Mitochondrial DNA 23: 187-200.
